# Clinical characteristics, triggering etiologies, and response of plasmapheresis in thrombotic microangiopathy in Taiwan

**DOI:** 10.1097/MD.0000000000025986

**Published:** 2021-05-21

**Authors:** Ching-Hu Chung, I-Jung Tsai, Min-Hua Tseng, Hsin-Hsu Chou, You-Lin Tain, Jeng-Daw Tsai, Yuan-Yow Chiou, Yee-Hsuan Chiou, Ching-Yuang Lin

**Affiliations:** a Department of Medicine, Mackay Medical College, New Taipei City; b Division of Nephrology, Department of Pediatrics, National Taiwan University Children Hospital, Taipei; c Division of Nephrology, Department of Pediatrics, Chang Gung Memorial Hospital, Taoyuan, Taiwan; d Department of Pediatrics, Xiamen Chang Gung Hospital, Ximen, China; e Department of Pediatrics, Ditmanson Medical Foundation Chia-Yi Christian Hospital, Chiayi; f Department of Bioinformatics and Medical Engineering, College of Information and Electrical Engineering, Asia University, Taichung; g Division of Pediatric Nephrology, Chang Gung Memorial Hospital-Kaohsiung Medical Center, Kaohsiung; h Division of Nephrology, Department of Pediatrics, MacKay Children's Hospital, Taipei; i Departments of Pediatrics, Institute of Clinical Medicine, National Cheng Kung University Medical College and Hospital, Tainan 704; j Department of Pediatrics, Kaohsiung Veterans General Hospital; k Department of Medical Technology, Fooyin University, Kaohsiung 831; l Clinical Immunological Center, Children's Hospital, China Medical University, Taichung, Taiwan.

**Keywords:** atypical hemolytic–uremic syndrome, complement dysregulation, plasmapheresis, thrombotic microangiopathy, thrombotic thrombocytopenic purpura

## Abstract

Thrombotic microangiopathy (TMA) syndromes are extraordinarily diverse in clinical presentations and etiologies. However, there are still a limited number of large cohort studies focusing on the underlying causes, outcomes, and response to plasmapheresis.

A retrospective study was designed to understand trigger etiologies, organ dysfunctions, clinical outcomes, and efficacy of plasmapheresis in patients with TMA. The whole population of Taiwan was set up into 2 cohorts: 875 patients with TMA in the 2006 cohort (2006–2010) and 1352 patients with TMA in the 2011 cohort (2011–2015). One hundred ninety-five patients in the 2006 cohort and 272 patients in the 2011 cohort were under plasmapheresis treatment.

The common underlying etiologies were pregnancy, followed by systemic lupus erythematosus, rheumatoid arthritis, transplantation and drugs, which were significantly higher than the control group. Stroke, seizure, arterial thrombosis, vascular stenosis, hypertension, myocardial infarction, and pancreatitis were the main clinical signs and extra-renal involvements. In the multivariate regression analysis, stroke, arterial thrombosis, peripheral arterial disease, and uremia were significantly higher compared with the control group. The mortality rate in TMA under plasmapheresis was significantly higher than all TMA cases (39.33% vs 15.39% in the 2006 cohort and 39.27% vs 15.06% in the 2011 cohort).

This study indicated the spectrum of underlying causes, extra-renal characteristics, and the response to plasmapheresis of patients with TMA in Taiwan. Of note, the poor clinical outcomes of plasmapheresis in patients with TMA might highlight the masked underlying etiology or worse disease condition that should be noticed.

## 1 Introduction

Thrombotic microangiopathy (TMA) is a potentially life-threatening disease characterized by endothelial damage, platelet aggregation into a thrombus, and an occlusion of the microvasculature.^[1]^ Its clinical manifestations include a variety of presentations, which include unexplained anemia, thrombocytopenia, kidney injury, unexplained neurologic findings, or other acute illnesses.^[2]^ However, the diagnosis is commonly inferred from the observation of microangiopathic hemolytic anemia and thrombocytopenia in an appropriate clinical setting. TMA includes thrombotic thrombocytopenic purpura (TTP; caused by a disintegrin and metalloproteinase with thrombospondin motifs 13 deficiency), Shiga toxin-mediated hemolytic–uremic syndrome (HUS; enterohaemorrhagic *Escherichia coli* possessing genes that encode the Shiga toxin), drug-induced TMA (DITMA), pregnancy-related TMA (P-TMA), autoimmune-related TMA, inborn error of vitamin B12 metabolism, and complement mediated-related TMA (CM-TMA; also known as atypical HUS [aHUS]).^[1,3,4]^ However, the prevalence and underlying etiologies of TMA in the Asian population remain unclear.

The complications of TMA are variable, ranging from vascular symptoms, acute kidney injury, gastrointestinal ischemia, pancreatitis, respiratory failure, visual disturbances, and neurologic deficit to cardiac involvement.^[2,5]^ Appropriate management of TMA mostly depends on uncovering the underlying etiologies, which are always unknown. The mortality rate was as high as 72% to 94% before the advent of effective therapy.^[3,4,8]^. It has been reported that therapeutic plasmapheresis reduces the mortality and is the mainstay of treatment for congenital and acquired TTP^[6–9]^; however, it is not effective in cases of other causes of TMA, such as CM-TMA.^[7,9,10]^

Several questions about TMA remain unanswered. First, a few studies have addressed the epidemiology, hence problems in the incidence and prevalence of TMA.^[11]^ Second, the main causes of TMA and their mortality are unclear.^[12,13]^ Third, the clinical manifestations of patients with TMA are also unclear. Therefore, this study aimed to investigate the incidence and prevalence, the etiology, the clinical presentations, the outcomes, and the response to plasmapheresis of patients with TMA. To investigate the underlying etiology, organ dysfunction, outcomes, and efficacy of plasmapheresis, the whole population from the National Health Insurance Research Database (NHIRD) between 2006 and 2015 was used to set up 2 cohorts (2006–2010 [2006 cohort] and 2011–2015 [2011 cohort]) for the diagnosis of TMA.

## 2 Materials and methods

### 2.1 Data source and patient definition

This is a retrospective, population-based, nationwide cohort study using claims records of the NHIRD between 2006 and 2015. Taiwan's National Health Insurance program was implemented in March 1995, and up to 99% of the country's 23 million residents receive medical care through this program. Due to the diagnosis and treatment may be changing over time between 2005 and 2015, we divide NHIRD into 2 cohorts (year 2005–2010 and year 2011–2015). The International Classification of Diseases, Ninth Revision, Clinical Modification code (ICD-9-CM code 446.6) was used to select the TMA. The detail patient selection procedure was shown in Supplemental Data 1. The Causes of Death Dataset (2006–2015) was used to estimate the patient survival status. To eliminate confounding factors and find the true risk of TMA, a case-matched control group with the same age/gender was selected, and 1:4 matching schemes were used.

### 2.2 Ethical approval of research

The protocol of this study was approved by the Joint Institutional Review Board of Taiwan R.O.C. (Protocol Number: 17-S-006-2).

### 2.3 Triggering/underlying conditions and clinical manifestations assessment

The diagnosis code used before the first TMA diagnosis was identified as triggering/underlying conditions. The diagnosis codes that were used after the first TMA diagnosis were identified as the clinical manifestations. These conditions were defined by the ICD-9 diagnosis codes, catastrophic illness certificate, or NHI codes. Coding used in this study was shown in Supplemental Data 1.

### 2.4 Concomitant medications used

The medications used for the treatment of lung cancer were in accordance with the ATC classifications.^[14]^ The anti-hypertensive drugs were identified by ATC code C02, corticosteroids were identified by ATC code H02A, immunosuppressive drugs were identified by ATC code L04, and anti-heart failure drugs were identified by ATC code C01.

### 2.5 Data analyses

SAS 9.1 (SAS Institute Inc., Cary, NC) was used for data analyses. The variable measures were identified based on the criteria described above. Categorical variables are presented as counts and percentages and were compared by Pearson's *χ*^2^ test or Fisher's exact test, as necessary. We adjusted for potential confounders using logistic regression models, and we reported the results as adjusted odds ratios (ORs) and 95% confidence intervals (CIs). The log-rank test was used to compare the Kaplan–Meier curves from control, all TMA and TMA treated with plasmapheresis. Statistical significance was set at *P* < .05.

## 3 Results

### 3.1 Sample description

**Figure 1:**
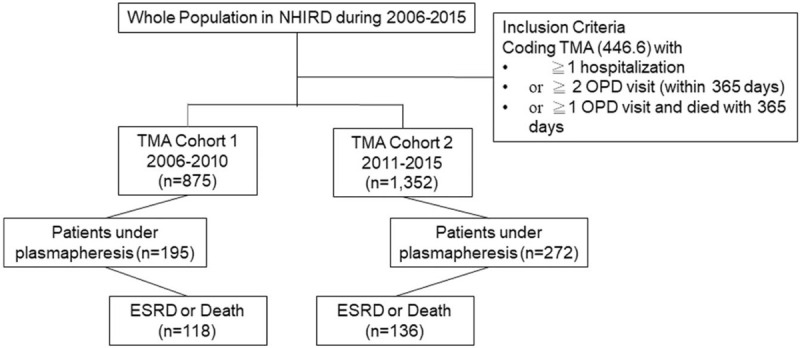
Selection and disposition of the study subjects.

We used the NHIRD from 2006 to 2015 to set up 2 cohorts, namely, the 2006 cohort and the 2011 cohort. Based on our inclusion criteria, there were 875 and 1352 patients with TMA in the 2005 cohort and the 2011 cohort (Fig. 1), respectively. Notably, 22.29% of patients (195 of 875) in the 2006 cohort and 20.12% of patients (272 of 1352) in the 2011 cohort underwent plasmapheresis treatment. The enrolled patients were predominantly female with a mean age of 52.90 ± 20.11 and 53.18 ± 18.58 years in the 2006 cohort and the 2011 cohort, respectively (Table 1). Our results indicated that the prevalence of TMA was the lowest in the age group of 0 to 20 years in both cohorts and the highest in the age group of 61 to 80 years in the 2006 cohort and 41 to 60 years in the 2011 cohort.

**Table 1:** Demographic data of the TMA patients.

	2006 cohort		2011 cohort	
	All TMA	TMA with plasmapheresis	All TMA	TMA with plasmapheresis
Patient number	875	195	1352	272
Gender				
Male	361 (41.26%)	74 (37.95%)	543 (40.16%)	116 (42.65%)
Female	509 (58.17%)	121 (62.05%)	806 (59.62%)	156 (57.35%)
Unknown	5 (0.57%)		3 (0.22%)	
Age				
0–20	52 (5.94%)	15 (7.69%)	66 (4.84%)	18 (6.61%)
21–40	194 (22.17%)	42 (21.54%)	284 (21.01%)	65 (23.90%)
41–60	275 (31.43%)	67 (34.36%)	528 (39.05%)	81 (29.78%)
61–80	297 (33.94%)	63 (32.31%)	375 (27.74%)	80 (29.41%)
≥81	57 (6.51%)	8 (4.10%)	99 (7.32%)	28 (10.30%)
Mean	52.9	52.04	53.18	53.42
STD	20.11	8.00	18.58	20.49

### 3.2 Underlying diseases in patients with TMA

We evaluated each patient's medical claims before the first TMA diagnosis and checked the difference between the patients with TMA and the control group to understand the underlying diseases or conditions that might cause TMA. The percentages of P-TMA, systemic lupus erythematosus (SLE), rheumatoid arthritis (RA), transplantation, drug-induced, and malignancy/anticancer therapy as the trigger or underlying conditions of TMA in both cohorts (all patients with TMA or patients with TMA under plasmapheresis) were significantly higher than those in the control group (Table 2). Patients with TMA under plasmapheresis were likely to be in worse condition, which may be associated with poor results. We found that patients with TMA under plasmapheresis exhibited a higher proportion of having the most triggering or underlying conditions than all patients with TMA. Of note, pregnancy was the most common underlying condition of patients with TMA under plasmapheresis in both cohorts.

**Table 2:** Underlying diseases in patients with TMA and plasmapheresis patients before the first TMA diagnosis.

	2006 cohort			2011 cohort		
Patient	Case-matched control (n = 3500)	TMA (n = 875)	TMA with plasmapheresis (n = 195)	Case-matched control (n = 5408)	TMA (n = 1352)	TMA with plasmapheresis (n = 272)
Pregnancy	184 (5.26%)	205 (23.43%)^‡^	102 (52.31%)^‡^	290 (5.36%)	280 (20.71%)^‡^	129 (47.43%)^‡^
SLE	8 (0.23%)	110 (12.57%)^‡^	28 (14.36%)^‡^	6 (0.11%)	118 (8.73%)^‡^	49 (18.01%)^‡^
Psoriatic arthritis	5 (0.14%)	≤3 (≤0.34%)	0 (0%)	10 (0.18%)	≤3 (≤0.22%)	≤3 (≤1.10%)
Ankylosing spondylitis	22 (0.63%)	18 (2.06%)^‡^	≤3 (≤1.54%)	38 (0.70%)	17 (1.26%)^‡^	≤3 (≤1.10%)
Rheumatoid arthritis	38 (1.09%)	48 (5.49%)^‡^	9 (4.62%)^‡^	79 (1.46%)	61 (4.51%)^‡^	12 (4.41%)^‡^
Psoriasis	19 (0.54%)	10 (1.14%)	≤3 (≤1.54%)	21 (0.39%)	12 (0.89%)^‡^	4 (1.47%)^‡^
Ulcerative colitis	≤3 (0.09%)	≤3 (≤0.34%)	0 (0%)	0 (0%)	≤3 (≤0.22%)	0 (0%)
Crohn's disease	26 (0.74%)	11 (1.26%)	≤3 (≤1.54%)	65 (1.2%)	23 (1.7%)	10 (3.66%)^‡^
Transplantation	≤3 (≤0.09%)	4 (0.46%)^‡^	≤3 (≤1.54%)^‡^	≤3 (≤0.06%)	12 (0.89%)^‡^	4 (1.47%)^‡^
Drug induced^§^	26 (0.74%)	37 (4.23%)^‡^	19 (9.74%)^‡^	106 (1.96%)	110 (8.14%)^‡^	51 (18.75%)^‡^
Malignancy/anticancer therapy	9 (0.26%)	10 (1.14%)^‡^	6 (3.08%)^‡^	20 (0.37%)	16 (1.18%)^‡^	7 (2.57%)^‡^

### 3.3 Multivariate regression analysis for underlying diseases of patients with TMA

Logistic regression revealed that patients were more likely to have TMA in both cohorts with pregnancy (adjusted OR in the 2006 cohort, 3.11; 95% CI, 2.45–3.96), ankylosing spondylitis (OR, 2.01; 95% CI, 1.02–3.93), RA (OR, 2.57; 95% CI, 1.38–3.69), SLE (OR, 36.88; 95% CI, 17.63–77.18), and DITMA (OR, 2.68; 95% CI, 1.41–5.11). Of note, the ORs of DI-TMA and P-TMA were significantly higher in patients with plasmapheresis than those without plasmapheresis in both cohorts (Table 3). On the contrary, the ORs in SLE patients with TMA under plasmapheresis (OR, 3.58; 95% CI 2.07–6.19 in the 2006 cohort; OR, 9.15; 95% CI, 5.69–14.71 in the 2011 cohort) were much lower in the total patients with TMA (OR, 36.88; 95% CI, 17.63–77.18 in the 2006 cohort; OR, 41.07; 95% CI, 18.88–89.36 in the 2011 cohort).

**Table 3:** Multivariate regression analyses for age, gender, and underlying diseases of TMA patients.

	2006 cohort				2011 cohort			
	TMA (N = 875)		TMA with plasmapheresis (N = 195)		TMA (N = 1352)		TMA with plasmapheresis (N = 272)	
	OR (95% CI)	*P* value	OR (95% CI)	*P* value	OR (95% CI)	*P* value	OR (95% CI)	*P* value
Age	1.00 (0.99–1.01)	.41	0.99 (0.98–1.00)	.23	0.99 (0.98–1.00)	.05	0.99 (0.98–1.00)	.07
Gender	1.09 (0.89–1.34)	.38	0.80 (0.56–1.15)	.3	1.09 (0.93–1.28)	.26	1.25 (0.94–1.68)	.13
Pregnancy	3.11 (2.45–3.96)	<.0001	8.39 (5.94–11.85)	<.0001	2.83 (2.32–3.45	<.0001	6.14 (4.59–8.20)	<.0001
Enterovirus	N.A.	N.A.	N.A.	N.A.	0 (0–∞)	.97	0 (0–∞)	.99
Psoriatic arthritis	0.82 (0.15–4.50)	.82	0 (0–∞)	.99	0.29 (0.04–2.01)	.21	0.21 (0.02–2.96)	.25
Ankylosing spondylitis	2.01 (1.02–3.93)	.043	0.73 (0.17–3.21)	.68	1.02 (0.54–1.94)	.95	0.63 (0.16–2.40)	.5
Rheumatoid arthritis	2.57 (1.38–3.69)	.001	0.81 (0.36–1.81)	.61	2.00 (1.38–2.89)	.0003	1.01 (0.50–2.04)	.97
Psoriasis	1.30 (0.58–2.93)	.52	1.84 (0.52–6.51)	.35	1.20 (0.54–2.66)	.65	1.38 (0.41–4.66)	.61
Ulcerative colitis	0.84 (0.06–11.90)	.9	0 (0–∞)	.99	∞ (0–∞)	.98	0 (0–∞)	.98
Crohn's disease	0.83 (0.38–1.78)	.62	0.18 (0.02–1.38)	.1	0.98 (0.59–1.64)	.95	2.14 (1.04–4.42)	.04
SLE	36.88 (17.63–77.18)	<.0001	3.58 (2.07–6.19)	<.0001	41.07 (18.88–89.36)	<.0001	9.15 (5.69–14.71)	<.0001
Transplantation	2.23 (0.34–14.77)	.41	0.96 (0.12–7.79)	.97	9.55 (1.16–78.59)	.04	0.86 (0.21–3.50)	.83
Drug induced^∗^	2.68 (1.41–5.11)	.003	4.10 (2.00–8.42)	.0001	2.12 (1.52–2.96)	<.0001	3.33 (2.20–5.04)	<.0001
Malignancy/anticancer therapy	2.10 (0.76–5.79)	.15	3.10 (1.02–9.41)	.047	1.58 (0.76–3.27)	.22	2.16 (0.86–5.42)	.1

### 3.4 The main clinical manifestations and extra-renal organ involvement

Because patients with TMA were associated with various diseases, we compared several diseases between the control group and patients with TMA in order to validate the differences. Patients with TMA were associated with a higher incidence of stroke, seizure, arterial thrombosis, vascular stenosis, hypertension, myocardial infarction, pancreatitis, and acute kidney injury than the control group in both cohorts (Table 4). Of note, TMA under plasmapheresis also indicated a higher percentage of the abovementioned complications than all patients with TMA.

**Table 4:** Clinical parameters and organ involvement of patients with TMA.

	2006 cohort			2011 cohort		
Patient	Case-matched control (n = 3500)	TMA (n = 875)	TMA with plasmapheresis (n = 195)	Case-matched control (n = 5408)	TMA (n = 1352)	TMA with plasmapheresis (n = 272)
CNS						
Stroke	279 (7.97%)	183 (20.91%)^‡^	52 (26.67%)^‡^	494 (9.13%)	242 (17.90%)^‡^	64 (23.53%)^‡^
Seizure	11 (0.31%)	33 (3.77%)^‡^	23 (11.79%)^‡^	24 (0.44%)	28 (2.07%)^‡^	13 (4.78%)^‡^
Heart						
Cardiomyopathy	6 (0.17%)	4 (0.46%)	≤3 (≤1.54%)^∗^	9 (0.17%)	5 (0.37%)	≤3 (≤1.10%)
Myocardial infarction	20 (0.57%)	15 (1.71%)^†^	5 (2.56%)^†^	32 (0.59%)	26 (1.92%)^‡^	13 (4.78%)^‡^
Hypertension	954 (27.26%)	393 (44.91%)^‡^	83 (42.56%)^‡^	1889 (34.93%)	557 (41.20%)^‡^	109 (40.07%)
Malignant hypertension	16 (0.46%)	13 (1.49%)^∗^	0 (0.00%)	35 (0.65%)	17 (1.26%)^∗^	≤3 (≤1.10%)
Gastrointestinal system						
Pancreatitis	23 (0.66%)	16 (1.83%)^†^	5 ((2.56%)^†^	35 (0.65%)	23 (1.70%)^‡^	10 (3.68%)^‡^
Colitis or gastroenteritis	680 (19.43%)	173 (19.77%)	35 (17.95%)	1204 (22.26%)	323 (23.89%)	37 (13.60%)^‡^
Diarrhea	47 (1.34%)	17 (1.94%)	5 (2.56%)	108 (2.00%)	38 (2.81%)	7 (2.57%)
Nausea or vomiting	164 (4.69%)	49 (5.60%)	8 (4.10%)	304 (5.62%)	119 (8.80%)^‡^	14 (5.15%)
Vessels						
Arterial thrombosis	11 (0.31%)	43 (4.91%)^‡^	5 (2.56%)^‡^	37 (0.68%)	40 (2.96%)^‡^	7 (2.57%)^†^
Vascular stenosis	7 (0.20%)	7 (0.80%)^‡^	≤3 (≤1.54%)^∗^	30 (0.55%)	12 (0.89%)	0 (0.00%)
Peripheral artery disease	39 (1.11%)	46 (5.26%)^‡^	≤3 (≤1.54%)	83 (1.53%)	63 (4.66%)^‡^	4 (1.47%)
Kidney						
ESRD	8 (0.23%)	24 (2.74%)^‡^	13 (6.67%)^‡^	9 (0.17%)	27 (2.00%)^‡^	16 (5.88%)^‡^

### 3.5 Multivariate regression analysis for main clinical signs and extra-renal involvement

Furthermore, the clinical manifestations of patients with TMA were investigated using logistic regression. The results revealed that patients with TMA in the 2006 cohort were more likely to have stroke (OR, 1.56; 95% CI, 1.19–2.05), seizure (OR, 5.15; 95% CI, 2.34–11.35), arterial thrombosis (OR, 8.74; 95% CI, 3.89–19.67), peripheral artery disease (OR, 2.28; 95% CI, 1.33–3.93), and acute kidney injury (OR, 1.09; 95% CI, 1.01–1.18), which was consistent with the 2011 cohort. By contrast, the OR of most diseases was higher, and the OR of RA was lower in patients with TMA under plasmapheresis than all patients with TMA. All these factors were calculated, and these data are presented in Table 5. End-stage renal disease (ESRD) was also common in both cohorts (Tables 4 and 5). The percentage of ESRD was higher in patients with TMA treated with plasmapheresis than others (6.67% vs 2.74% in the 2006 cohort and 5.88% vs 2.00% in the 2011 cohort).

**Table 5:** Multivariate regression analysis for clinical parameters and organ involvement in TMA patients.

	2006 cohort				2011 cohort			
	TMA (N = 875)		TMA with plasmapheresis (N = 195)		TMA (N = 1352)		TMA with plasmapheresis (N = 272)	
	OR (95% CI)	*P* value	OR (95% CI)	*P* value	OR (95% CI)	*P* value	OR (95% CI)	*P* value
CNS								
Stroke	1.56 (1.19–2.05)	.002	1.96 (1.28–2.99)	.002	1.70 (1.38–2.11)	<.0001	2.14 (2.13–2.16)	<.0001
Seizure	5.15 (2.34–11.35)	<.0001	18.02 (8.40–38.66)	<.0001	1.82 (0.95–3.47)	.07	5.24 (5.15–5.33)	<.0001
Heart								
Cardiomyopathy	0.94 (0.18–4.87)	.94	0.84 (0.05–14.29)	.9	1.58 (0.51–4.90)	.43	4.26 (4.11–4.41)	<.0001
Myocardial infarction	1.21 (0.53–2.73)	.65	2.03 (0.64–6.41)	.23	1.64 (0.91–2.95)	.1	6.65 (6.54–6.75)	<.0001
Hypertension	1.16 (0.91–1.49)	.24	0.72 (0.48–1.08)	.11	0.78 (0.65–0.95)	.01	0.62 (0.61–0.63)	<.0001
Malignant hypertension	2.14 (0.91–5.04)	.08	0 (0–∞)	.98	1.25 (0.62–2.52)	.53	0.53 (0.52–0.55)	<.0001
ECMO	∞ (0–∞)	.99	∞ (0–∞)	.99	∞ (0–∞)	.96	∞ (0–∞)	.55
Gastrointestinal system								
Pancreatitis	1.44 (0.62–3.34)	.4	3.18 (1.12–9.04)	.03	1.85 (0.98–3.48)	.06	4.61 (4.53–4.70)	<.0001
Colitis or gastroenteritis	0.56 (0.44–0.72)	<.0001	0.53 (0.35–0.82)	.005	0.73 (0.61–0.87)	.0006	0.44 (0.43–0.45)	<.0001
Diarrhea	0.91 (0.45–1.84)	.8	1.04 (0.31–3.45)	.95	1.02 (0.66–1.59)	.92	1.09 (1.07–1.11)	<.0001
Nausea or vomiting	0.70 (0.46–1.06)	.09	0.38 (0.16–0.89)	.026	1.15 (0.87–1.52)	.34	0.64 (0.63–0.65)	<.0001
Vessels								
Arterial thrombosis	8.74 (3.89–19.67)	<.0001	1.00 (0.32–3.14)	.99	2.17 (1.24–3.79)	.007	0.85 (0.83–0.87)	<.0001
Vascular stenosis	0.99 (0.29–3/45)	.99	0 (0–∞)	.98	0.64 (0.29–1.43)	.28	0.17 (0.16–0.17)	<.0001
Peripheral artery disease	2.28 (1.33–3.93)	.003	0.27 (0.07–0.99)	.048	1.66 (1.10–2.51)	.016	0.44 (0.43–0.45)	<.0001
Kidney								
ESRD	1.09 (1.01–1.18)	.037	1.14 (1.05–1.23)	.001	1.52 (1.22–1.89)	.0001	2.22 (2.21–2.23)	<.0001

### 3.6 Concomitant medications in patients with TMA

Medication use was also determined in this study to confirm the signs and diseases associated with patients with TMA. The results were consistent with the clinical manifestations determined by the International Classification of Diseases, Ninth Revision, codes. Anti-hypertension drugs, corticosteroids, immunosuppressive, and anti-heart failure drugs were significantly more frequently prescribed in patients with TMA than patients in the control group (Table 6). Medication prescription was significantly higher in patients with TMA under plasmapheresis than the total patients with TMA.

**Table 6:** Medication used in TMA patients.

	Total	Anti-hypertensive agents	Corticosteroid	Intravenous immunoglobulin	Immunosuppressants	Medications for heart failure
2006 cohort						
Control	3500	218 (6.23%)	1037 (29.63%)	0 (0%)	8 (0.23%)	66 (1.89%)
TMA	875	127 (14.51%)^∗^	583 (66.63%)^∗^	10 (1.14%)^†^	98 (11.20%)^∗^	39 (4.46%)^∗^
TMA with plasmapheresis	195	38 (19.49%)^∗^	171 (87.69%)^∗^	5 (2.56%)^∗^	32 (16.41%)^∗^	6.67%)^∗^
2011 cohort						
Control	5408	309 (5.71%)	2063 (38.15%)	≦3 (≦0.06%)	18 (0.33%)	74 (1.37%)
TMA	1352	166 (12.28%)^∗^	925 (68.42%)^∗^	16 (1.18%)^∗^	140 (10.36%)^∗^	48 (3.55%)^∗^
TMA with plasmapheresis	272	56 (20.59%)^∗^	269 (95.22%)^∗^	10 (3.68%)^∗^	46 (16.91%^∗^	12 (4.41%)^∗^

### 3.7 Clinical outcome of patients with TMA

**Figure 2:**
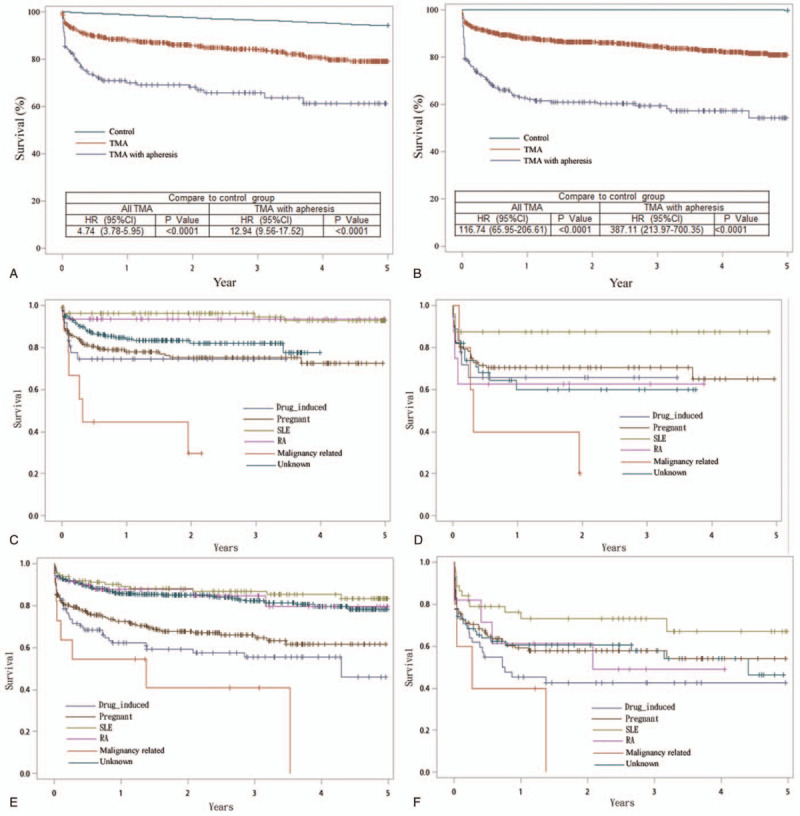
Kaplan–Meier curves of survival rates. The survival curves among all TMA, TMA with plasmapheresis, and control group of (A) 2006 cohort, (B) 2011 cohort were shown. The survival curves among different causes of TMA patients for (C) 2006 cohort in all TMA patients, (D) 2006 cohort in TMA patients with plasmapheresis, (E) 2011 cohort in all TMA patients, (F) 2011 cohort in TMA patients with plasmapheresis were shown. TMA exclude drug induced, pregnant, SLE, RA, and malignancy related TMA were classified as other. RA = rheumatoid arthritis, SLE = systemic lupus erythematosus, TMA = thrombotic microangiopathy.

Because inappropriately treating patients with TMA may be associated with fatal outcome, we analyzed the mortality rate of all the patients with TMA and those treated with plasmapheresis by linking each patient's survival data. The survival of all patients with TMA in the 2006 cohort (hazard ratio [HR], 4.74; 95% CI, 3.78–5.95) and the 2011 cohort (HR, 116.74; 95% CI, 65.95–206.61) was significantly lower than that of the control group (log-rank test, *P* < .001) (Fig. 2A and B). In patients with TMA under plasmapheresis, a significant difference was found on mortality between the 2006 cohort (HR, 12.94; 95% CI, 9.56–17.52) and the 2011 cohort (HR, 387.11; 95% CI, 213.97–700.35). Supplemental Data 2 presents the annual mortality rates in each year. The 5-year mortality rate was significantly higher in patients with TMA under plasmapheresis (39.33% in the 2006 cohort and 39.27% in the 2011 cohort) than that in the total patients with TMA (15.39% in the 2006 cohort and 15.06% in the 2011 cohort).

Furthermore, we sub-analyzed the causes of mortality to investigate the causes of TMA and the association with overall survival in patients with TMA. Among these causes, malignancy-associated TMA had the worst survival, followed by DITMA (Fig. 2C–F). Compared with the TMA with other causes, the HRs of malignancy-related TMA (HR, 5.24; 95% CI, 2.40–11.44), DITMA (HR, 3.06; 95% CI, 2.08–4.51), and P-TMA (HR, 2.28; 95% CI, 1.65–3.16) in the 2011 cohort were significantly higher (Supplemental Data 3). In patients with TMA under plasmapheresis in both cohorts, no significant difference was found in each group in comparison with the other group.

## 4 Discussion

To the best of our knowledge, this is the first retrospective cohort study that examined the etiologies, clinical manifestations, response to plasmapheresis, and mortality using a nationally representative sample. The prevalence rates of TMA in Taiwan and other countries were similar.^[15–17]^ Pregnancy was the leading underlying cause of TMA in our cohort study. Furthermore P-TMA is the highest cause in all TMA (23.43% in the 2006 cohort and 20.71% in the 2011 cohort) as well as in patients with TMA treated with plasmapheresis (52.31% in the 2006 cohort and 47.43% in the 2011 cohort). In fact, P-TMA has been reported to account for 8% to 20% of all TMA cases.^[15–17]^ Pregnancy increases the risk of a wide spectrum of TMA ranging from TTP to HUS.^[18,19]^ In addition, pregnancy may also increase the risk of relapse in patients from acquired and autoimmune TTP.^[20]^ In the management of pregnancy-associated HUS, Bruel et al reported that plasmapheresis did not improve the renal outcome of pregnancy-associated HUS, and the outcome of ESRD was high in patients with or without plasmapheresis.^[21]^ In addition, in this study, pregnancy-related HUS is associated with genetic variants in complement genes in 56% of patients, which might be the cause of poor treatment response to plasmapheresis which is the mainstay treatment of pregnancy-related TTP.^[22]^ Moreover, SLE-related TMA were the second most common cause found in all patients with TMA as well as in patients with TMA under plasmapheresis in both cohorts. A previous study has reported that SLE has a prevalence of around 30% of TMA,^[23]^ which is higher compared with our results. These differences may be caused by the different methods used for case selection. Most studies on TMA enrolled less than 100 patients and predicting the proportion from each of the causes was difficult in a small population cohort. However, Chen et al reported that infection is a major risk factor that triggers TMA in patients with SLE in Taiwan, and plasmapheresis is an alternative treatment modality.^[24]^ Sun et al also reported that rituximab improves the survival in SLE-induced TMA instead of plasmapheresis.^[25]^ Therefore, plasmapheresis may not be an effective treatment choice for SLE-induced TMA.

Plasmapheresis started when TMA was suspected in Taiwan. Plasmapheresis is not only used as the first-line therapy but is also restarted in every relapse or exacerbation of TMA.^[26]^ Plasmapheresis is started because of worsening of clinical conditions or because the diagnosis is not yet available, PE is generally continued until the results of ADAMTS13 activity testing become available. Aside from the clear benefit of plasmapheresis in patients with TMA caused by TTP, the overall response of plasmapheresis in our patients with TMA is poor including higher mortality and ESRD. aHUS is characterized by pathologic complement activation, resulting in systemic endothelial and organ damage, which is currently emergent and contributes to devastating outcome in spite of plasmapheresis treatment.^[27,28]^ The general diagnosis of aHUS should exclude Shiga toxins and TTP.^[29]^ Because the NHIRD lacks clinical and laboratory data, it is difficult to differentiate aHUS from other causes of TMA. aHUS was considered to be one of the unrecognized underlying diseases and thus may contribute to the limited response of plasmapheresis and poor prognosis before the era of anti-C5 therapy. In our study, aHUS should raise our attention in those patients with TMA with poor response to plasmapheresis. In the age of 50 years, age-specific survival in patients with ESRD was reported to decrease from 20.2 life-years lost to 23.0 life-years lost in 1977 to 2007 compared with the general population.^[30]^ This may be another reason for poor clinical outcomes in patients with TMA under plasmapheresis treatment.

Acquired TTP, DITMA, or hereditary CM-TMA is more commonly presented in adults.^[2]^ Although immune-mediated reactions and direct toxic reactions are 2 major mechanisms involved in DITMA, the mechanisms of DITMA in most drugs are still unknown.^[31]^ The treatment for DITMA is to discontinue the drug immediately, and no standardized modalities or treatments have been established so far.^[32]^ Furthermore, the reasons of higher mortality in patients with DITMA might be caused by medications, such as calcineurin inhibitors, which are commonly used in patients with transplantations.^[33–35]^ Due to the lack of high-quality evidence for the benefit of plasmapheresis in DITMA, plasmapheresis is not recommended by the American Society for Apheresis.^[9]^ However, DITMA may improve after drug adjustment.

Our study reveals that the OS was worse in malignancy-related TMA compared with other causes of TMA (Fig. 2C–F). Because malignancy-related mortality was higher than the general population with or without TMA, poor OS in malignancy-related TMA is not surprising.^[36]^ Malignancy-related TMA was also reported with the highest mortality rates of 10% to 40% and in some cases up to 60% to 70%.^[37]^ Among these causes, DITMA came with the second highest mortality rate among the different causes of TMA. P-TMA had the third place in mortality rate among these TMA. P-TMA mostly happened in the postpartum period. Although P-TMA is a rare condition and associated with poor maternal outcomes, there were very limited studies that investigate the maternal mortality. Our study was one of few studies to demonstrate that P-TMA was life threatening, and mortality rate was even higher than RA- or SLE-associated TMA.

Some limitation should be considered by using insurance claims data, including coding errors, omissions, or incomplete data. Because this is a big data analysis, selection bias of patients should be minimised compared with single-centre studies. This study has several limitations. First, this may lead to the inability of estimating self-payment for medications, laboratory data (ADAMTS13 activity test, etc), and detailed patient information (height, weight, etc). Second, multiple diseases and multiple diagnoses may influence the patient's classification and their outcome. Third, the coding of TMA may differ with different hospitals or different physicians. Therefore, due to protection of their personal privacy, the proportion of our subjects may not be correct. Finally, the Health and Welfare Data Science Center does not allow exporting results that are equal to or less than 2 cases. Because TMA are rare diseases, some results could not reflect the exact numbers.

This study has attempted to shed some light in the understanding of triggering etiologies, extra-renal involvement, and efficacy of plasmapheresis for TMA syndrome in Taiwan. These results will help the clinicians in considering the etiologies as well as help them during TMA diagnosis and management. Further prospective randomized studies are needed to verify our findings, which might improve the patients’ outcome by using effective treatments earlier and decrease mortality and long-term morbidities.

## Author contributions

Lin CY designed and supervised the study. Chung CC collected and analyzed the data. Chung CC, Tsai IJ, Tseng MI, Chou HH, Tain YL wrote the manuscript. Tasi JD, Chiou YY, Chiou YH critically reviewed. All authors read and approved the final manuscript.

**Conceptualization:** Ching-Hu Chung, Ching-Yuang Lin.

**Data curation:** Ching-Hu Chung, Ching-Yuang Lin.

**Formal analysis:** Ching-Hu Chung.

**Investigation:** Ching-Hu Chung, I-Jung Tsai, Min-Hua Tseng, Hsin-Hsu Chou, You-Lin Tain, Jeng-Daw Tsai, Yuan-Yow Chiou, Yee-Hsuan Chiou.

**Methodology:** I-Jung Tsai, Hsin-Hsu Chou, You-Lin Tain, Jeng-Daw Tsai, Yuan-Yow Chiou, Yee-Hsuan Chiou.

**Supervision:** Ching-Yuang Lin.

**Validation:** Ching-Yuang Lin.

**Writing – original draft:** Ching-Hu Chung.

**Writing – review & editing:** I-Jung Tsai, Min-Hua Tseng, Hsin-Hsu Chou, You-Lin Tain, Jeng-Daw Tsai, Yuan-Yow Chiou, Yee-Hsuan Chiou, Ching-Yuang Lin.

## Supplementary Material

Supplemental Digital Content

## Supplementary Material

Supplemental Digital Content

## Supplementary Material

Supplemental Digital Content
