# Supplementary material for: Clinical characteristics, triggering etiologies, and response of plasmapheresis in thrombotic microangiopathy in Taiwan
Source: Medicine (Baltimore). 2021 May 21;100(20):e25986. doi: 10.1097/MD.0000000000025986 (PMC8137071; doi:10.1097/MD.0000000000025986)
Supplement: Supplemental Digital Content [file medi-100-e25986-s001.docx]

Supplement data 1

**Patient Definition**

Patients were classified as having TMAs and were included in the analysis if the diagnostic code for TMA was obtained during at least one hospital admission or during more than two outpatient visits within 365 calendar days, or during one outpatient visit but the patient died within 365 calendar days. Patients with TMA who were treated with plasmapheresis (ICD-9-CM procedure code 99.71 or NHI code 58008C; transfusion of blood and blood components: ICD-9 procedure-code 99.0 or NIH code 93013C、93021C) were selected as those with severe TMA.

**Triggering/underlying conditions Assessment.**

(1) pregnancy: patients with prenatal visit; (2) systemic lupus erythematosus (SLE): ICD-9 diagnosis code 710 and with catastrophic illness certificate (ICD-9 code: 710); (3) psoriatic arthritis (PA): ICD-9 diagnosis code 713 and with catastrophic illness certificate (ICD-9 code: any); (4) ankylosing spondylitis (AS): ICD-9 diagnosis code 720 and with catastrophic illness certificate (ICD-9 code: any); (5) rheumatoid arthritis (RA): ICD-9 diagnosis code 714 and with catastrophic illness certificate (ICD-9 code: 714); (6) psoriasis: ICD-9 diagnosis code 696.1 and with catastrophic illness certificate (ICD-9 code: any); (7) ulcerative colitis: ICD-9 diagnosis code 556.9 and with catastrophic illness certificate (ICD-9 code: 556.9); (8) Crohn’s disease: ICD-9 diagnosis code 555 and with catastrophic illness certificate (ICD-9 code: 555); (9) organ transplantation: ICD-9 diagnosis code V42; (10) DITMA: calcineurin inhibitors, cyclosporine/tacrolimus, quetiapine, quinine and VEGF used; and (11) malignancy/anticancer therapy: antineoplastic agents (Anatomical Therapeutic Chemical [ATC] code: L01) used.

**Clinical manifestations Assessment.**

(1) stroke: ICD-9 diagnosis codes 430–432 and 433–438; (2) seizure: ICD-9 diagnosis code 780.3; (3) arterial thrombosis: ICD-9 diagnosis code 444; (4) vascular stenosis: ICD-9 diagnosis code 433.1; (5) hypertension: ICD-9 diagnosis codes 401–405; (6) malignant hypertension: ICD-9 diagnosis code 401.0; (7) cardiomyopathy: ICD-9 diagnosis code 425; (8) myocardial infarction: ICD-9 diagnosis code 410; (9) pancreatitis: ICD-9 diagnosis code 557.0-1; (10) colitis or gastroenteritis: ICD-9 diagnosis code 558.9; (11) diarrhea: ICD-9 diagnosis code 787.91; (12) nausea or vomiting: ICD-9 diagnosis code 787.0; (13) peripheral artery disease: ICD-9 diagnosis code 443.9; (14) end-stage renal disease (ESRD): catastrophic illness certificate code (ICD-10: N18.5, N18.6, I12.0, I13.11 and I13.2; ICD-9: 585, 40301, 40311, 40391, 40402, 40403, 40412, 40413, 40492 and 40493); and (15) extracorporeal membrane oxygenation (ECMO): NIH code 39.65.
