# Supplementary material for: Clinical characteristics, triggering etiologies, and response of plasmapheresis in thrombotic microangiopathy in Taiwan
Source: Medicine (Baltimore). 2021 May 21;100(20):e25986. doi: 10.1097/MD.0000000000025986 (PMC8137071; doi:10.1097/MD.0000000000025986)
Supplement: Supplemental Digital Content [file medi-100-e25986-s002.docx]

Supplement Table 2. The mortality rate of TMA patients.

| **2006 Cohort** |  |  |  |  |  |
| --- | --- | --- | --- | --- | --- |
| **Annual Mortality rate** | **1st** | **2nd** | **3rd** | **4th** | **5th** |
| Control | 1.29% | 2.46% | 3.51% | 4.66% | 5.91% |
| All-TMA | 11.16% | 12.69% | 13.63% | 14.69% | 15.39% |
| TMA with apheresis | 31.46% | 34.83% | 35.96% | 38.20% | 39.33% |
| **2011 Cohort** |  |  |  |  |  |
| **Annual Mortality rate** | **1st** | **2nd** | **3rd** | **4th** | **5th** |
| Control | 0.00% | 0.00% | 0.00% | 0.00% | 0.30% |
| All-TMA | 11.28% | 12.41% | 13.40% | 14.23% | 15.06% |
| TMA with apheresis | 34.82% | 35.63% | 36.44% | 37.25% | 39.27% |
