# Supplementary material for: Clinical characteristics, triggering etiologies, and response of plasmapheresis in thrombotic microangiopathy in Taiwan
Source: Medicine (Baltimore). 2021 May 21;100(20):e25986. doi: 10.1097/MD.0000000000025986 (PMC8137071; doi:10.1097/MD.0000000000025986)
Supplement: Supplemental Digital Content [file medi-100-e25986-s003.docx]

Supplement Data 3. The survival odds ratio of TMA patients.

|  | **2006 Cohort** | | | | **2011 Cohort** | | | |
| --- | --- | --- | --- | --- | --- | --- | --- | --- |
|  | **Compare to other TMA** | | | | **Compare to other TMA** | | | |
|  | **All TMA** | | **TMA with plasmapheresis** | | **All TMA** | | **TMA with plasmapheresis** | |
|  | **P value** | **OR (95%CI)** | **P value** | **OR (95%CI)** | **P value** | **OR (95%CI)** | **P value** | **OR (95%CI)** |
| **Drug-induced** | 0.15 | 1.72 (0.83-3.59) | 0.87 | 0.92 (0.36-2.41) | <0.001 | 3.06 (2.08-4.51) | 0.21 | 1.40 (0.82-2.39) |
| **Malignancy related** | <0.001 | 5.06 (2.12-12.07) | 0.19 | 2.11 (0.69-6.41) | <0.001 | 5.24 (2.40-11.44) | 0.11 | 2.33 (0.82-6.64) |
| **Pregnant** | 0.10 | 1.45 (0.93-2.27) | 0.46 | 0.79 (0.42-1.49) | <0.001 | 2.28 (1.65-3.16) | 0.98 | 0.99 (0.63-1.57) |
| **RA** | 0.86 | 0.36 (0.11-1.17) | 0.90 | 1.08 (0.31-3.76) | 0.91 | 0.96 (0.48-1.93) | 0.86 | 1.09 (0.42-2.81) |
| **SLE** | 0.0049 | 0.29 (0.12-0.68) | 0.052 | 0.29 (0.08-1.02) | 0.34 | 0.76 (0.44-1.32) | 0.099 | 0.57 (0.29-1.11) |
